# Functional Expression Study of *Igf2* Antisense Transcript in Mouse

**DOI:** 10.1155/2014/390296

**Published:** 2014-01-16

**Authors:** Carolina Duart-Garcia, Martin H. Braunschweig

**Affiliations:** ^1^Institute of Genetics, Vetsuisse Faculty, University of Bern, Bremgartenstrasse 109a, 3001 Berne, Switzerland; ^2^Graduate School for Cellular and Biomedical Sciences, Theodor Kocher Institute, University of Bern, Freiestrasse 1, 3012 Berne, Switzerland

## Abstract

Insulin-like growth factor antisense gene (*Igf2as*) expression was investigated in different mouse tissues during development, in differentiating C2C12 cells and in a ΔDMR1-U2 knockout mouse model. The expression levels of *Igf2as* were high in fetal and newborn liver and muscle tissues compared to adults. The *Igf2as* gene was also expressed in placenta and in brain. The expression data suggests that the *Igf2as* gene plays a role in early development of the mouse and in placenta. There was no consistent evidence for an interaction between *Igf2* and *Igf2as* transcripts. Furthermore, in knockout placentas lacking *Igf2as* transcription, *Igf2* expression was comparable to that in wild type. These results indicate that *Igf2as* does not regulate *Igf2* sense transcripts. In previous studies, it was suggested that the ΔDMR1-U2 knockout mouse showing intrauterine growth restriction was caused by the absence of placenta-specific *Igf2* P0 transcription. We conclude that the ΔDMR1-U2 deletion phenotype should be reconsidered in the light of a functional *Igf2as* gene.

## 1. Introduction

The insulin-like growth factor 2 gene (*Igf2*) is an imprinted gene, paternally expressed and encodes for the insulin-like growth factor II peptide [1, 2]. In mice the *Igf2* gene has five promoters, from which three main transcripts, a placenta specific transcript and a newly described mesoderm-specific transcript, originate [3]. Moore et al. [4] described multiple imprinted sense and antisense transcripts from the *Igf2* locus. The *Igf2as* gene located within *Igf2* is transcribed from the complementary DNA strand. We recently found that *Igf2as* transcripts are located in the cytoplasm and associated with polysomes indicating a protein coding function [5].

To further elucidate the function of *Igf2as*, we investigated the ΔDMR1-U2 knockout mouse [6]. This mouse has a 5 kb deletion within the *Igf2* gene comprising DMR1, an adjacent repeat sequence mostly embedded in exon U2 of the placenta specific *Igf2* P0 transcript and *Igf2as* transcripts. The maternal transmission of the ΔDMR1-U2 deletion results in loss of *Igf2* imprinting in heart, kidney, and lung without affecting *H19* gene expression [6]. The authors conclude that their result demonstrates that DMR1 plays a role in *Igf2* imprinting regulation and gene expression independent of *H19* [6]. In that study, paternal transmission of the ΔDMR1-U2 deletion was associated with intrauterine growth restriction (IUGR) manifested by mutants birth weight being 71% that of normal mice. Later, Constância et al. [7] reported that the 5 kb ΔDMR1-U2 deletion abolishes expression of the *Igf2* P0 transcript in the labyrinthine trophoblast of the placenta where it is specifically expressed. Noteworthy, placental growth deficiency in ΔDMR1-U2 placentas resembled that of *Igf2*-null mice lacking IGF-II peptide in all placental layers and the fetus which was unexpected since P0 transcripts constitute about 10% of total *Igf2* transcripts in placenta [8]. Constância et al. [7] suggest that differential translatability of *Igf2 *transcripts may explain this observation. However, the lower birth weight of mutant ΔDMR1-U2 pups was compensated during the first three months of life. They could also demonstrate with inert hydrophilic molecules that passive permeability of the mutant placenta was reduced compared to the wild type. However, they showed that the mutant placenta actively transferred more than wild-type placenta indicating compensatory effects to the passive permeability and the smaller placenta size.

The permeability of the mouse placenta to hydrophilic solutes was further studied in ΔDMR1-U2 mice versus wild-type mice [9]. In their study they found that the permeability for hydrophilic solutes was significantly reduced in the ΔDMR1-U2 knockout placenta at E19. Stereological analysis showed a reduction in surface area and an increase in thickness of the exchange barrier in the ΔDMR1-U2 knockout labyrinthine layer of the placenta. This suggests that labyrinthine P0 *Igf2* expression has a function in the development of normal diffusional exchange characteristics of the mouse placenta influencing fetal growth [9]. Constância et al. [10] further investigated the placental nutrient supply and the fetal demand in the ΔDMR1-U2 deletion mouse model (*Igf2* P0^+/−^) and an *Igf2*-null mouse model [10]. Constância et al. [10] could further show by using *Igf2* P0^+/−^ and *Igf2*-null mice models that placental nutrient transfer occurs in response to fetal nutrient demands and involves *Igf2* mediation. Additional evidence for the placental adaption was provided by showing lower expression of the transplacental calcium transfer protein calbindin-D9K in *Igf2* P0^+/−^ at E17 but not at E19 compared to that of wild types [11]. More recently, it was shown that adult *Igf2* P0^+/−^ mice but not *Igf2*-null mice exhibited behavioral phenotypes [12]. These mice were exposed to a wide range of emotion-related behaviors tests and *Igf2* P0^+/−^ mice showed increased reactivity to acute anxiety-including stimuli. It was further found that anxiety associated genes in the hippocampus of these mice were altered. These long-term behavioral effects were attributed to the imbalance between fetal demand and placental supply of nutrients during gestation [13].

In this study, we performed an expression analysis of *Igf2* and *Igf2as* transcripts in ΔDMR1-U2 and wild-type placentas from different development stages. The expression of these genes is studied in wild type placentas and in ΔDMR1-U2 placentas which are lacking *Igf2* P0 and *Igf2as* expression. Our results from this comparison are challenging previous findings from this mouse model. We also followed the expression patterns of this sense/antisense pair in different tissues during development and measured *Igf2/Igf2as* expression in differentiating C2C12 cells for functional characterization of the putative protein coding *Igf2as *gene.

## 2. Material and Methods

### 2.1. Cell Culture

C2C12 murine myoblast cells were grown as described previously [5]. The cells were grown to 100% confluence and changed into a differentiation medium (Dulbecco's Modified Eagle's Medium supplemented with 2% horse serum and 1% penicillin-streptomycin solution). The differentiation media were replaced daily during a period of 6 days. At each time point, the cells of three culture flasks were harvested separately, RNA isolated, and used for quantification by real-time PCR.

### 2.2. Animals

The experiments were carried out in strict accordance with Swiss Federal Law on Animal Protection of 16 December 2005 (Tierschutzgesetz TSchG, SR 455), Art. 32, Absatz 1; Ordinance on Animal Protection of 23 April 2008 (Tierschutzverordnung TSchV, SR 455.1). Brain, muscle and liver tissues were collected from wild-type C57BL/6 fetuses at E18 (embryonic day 1 (E1) = day of plug), from newborn and adult mice. Also brain samples were collected from embryos at E14. ΔDMR1-U2 transgenic male mice carrying the mutation on the paternal allele were donated from M.R. Dilworth and colleagues [6, 11]. The males containing the mutation were crossed with wild-type C57BL/6 females. Mice resulting from these crossings were sacrificed at E12, E14, E16, and E19 and placentas were collected. DNA from each mouse tail was extracted to genotype for the ΔDMR1-U2 deletion. The primers used for this PCR test are shown in Table 1. From the crosses between male carriers of the ΔDMR1-U2 deletion and C57BL/6 females, three mutant placentas and three wild-type littermates were used for the analysis.

### 2.3. RNA Extraction and Quantitative Real-Time PCR (qRT-PCR)

**Figure 1:**
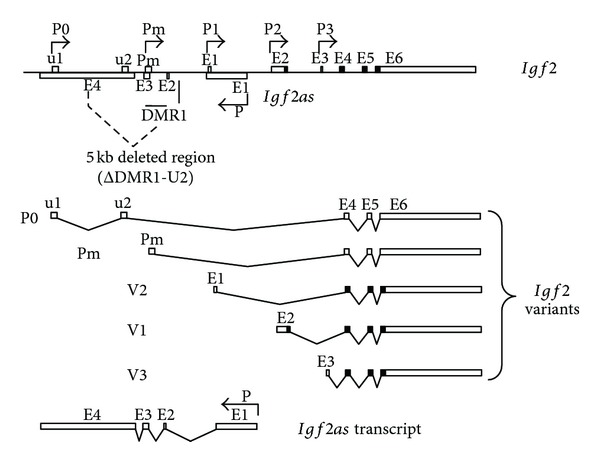
Structure of *Igf2 *gene, *Igf2as*, and ΔDMR1-U2 mutation. The arrows indicate the five promoters (P0, Pm, P1, P2, and P3) for *Igf2 *and(P) for *Igf2as*. DMR1 indicates the position of the DMR1 regulatory region. V1, V2, V3, P0, and Pm indicate the five variants of *Igf2 *gene. The 5 kb deleted region in ΔDMR1-U2 mice is indicated.

RNA extraction was performed using Trizol reagent according to manufacturer's protocol (Invitrogen). All RNA samples were treated with DNase I (Ambion). The RNA was further purified with the RNeasy Mini Kit (Qiagen). The cDNA was synthesized by reverse transcription of 1 *μ*g total RNA using the QuantiTect Reverse Transcription kit according to the manufacturer's protocol (Qiagen). Each sample was quantified in triplicates for all *Igf2 *variants (V1, V2, V3, and Pm) and the *Igf2as* transcript by qPCR using TaqMan probes or conjugated minor groove binder (MGB) probes to measure *Igf2as* transcription (Table 2) (Figure 1). The Ct values of the target were normalized by subtracting the mean of the Ct values from *Actb* and *Gapdh*. The normalized ΔCt values were converted to relative expression. The relative quantifications were calculated relative to the first value which was set to 1 in each respective figure. The calculations were performed for each of three samples measured in triplicate, averaged, and had the standard deviations calculated. The normalized data was analyzed by using two-tailed Student's *t*-test.

## 3. Results

### 3.1. *Igf2* and *Igf2as* Expressions in Different Tissues during Development

**Figure 2:**
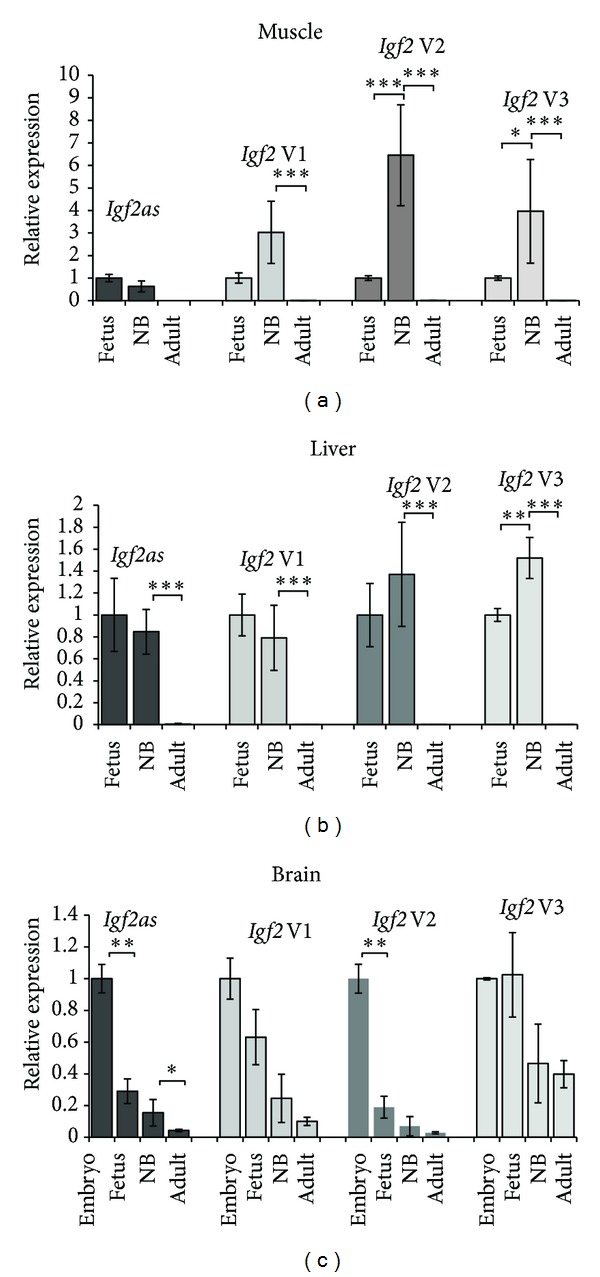
Quantification of expression levels of *Igf2 *variants and *Igf2as *transcripts in different tissues. *Igf2* variants and *Igf2as* were quantified in muscle (a), liver (b), and brain (c) tissues of fetus, newborn (NB), and adult and included embryonic brain. V1, V2, V3, and *Igf2as* stand for *Igf2* variants 1, 2, 3, and *Igf2 *antisense, respectively. The results are expressed in relative expression, calculated relative to the first value. Each bar represents the mean of three samples each analyzed in triplicate. The standard deviations are indicated as error bars. *P < 0.05, **P < 0.01, and ***P < 0.001.

We found relatively high *Igf2* and *Igf2as* gene expression in fetal and newborn liver and muscle tissue samples and a significant downregulation of these genes in adult tissues (Figures 2(a) and 2(b)). All three *Igf2 *variants followed similar pattern of expression at fetal, newborn, and adult stages of development. In muscle, we found comparable expression levels of *Igf2as* transcripts in fetus and newborn whereas the expression of *Igf2* variants increased in newborn (Figure 2(a)). *Igf2as* and *Igf2* variants were significantly downregulated in adult tissues (Figures 2(a) and 2(b)). *Igf2as *levels were not detectable after 40 qPCR cycles in adult muscle tissue. In liver, the level of *Igf2as* and *Igf2* expressions is similar in fetus and newborn, except for variant 3 which significantly increased in newborn (Figure 2(b)). Transcripts originating from the Pm promoter were expressed at very low levels (data not shown). In brain we found significantly higher expression of *Igf2 *variant 2 and *Igf2as* in embryos compared to fetus with a continuous down-regulation towards the adult stage (Figure 2(c)). *Igf2* variants and *Igf2as* followed this pattern, except variant 3 (Figure 2(c)). Pm transcripts in brain were not detected after 40 qPCR cycles.

### 3.2. *Igf2* and *Igf2as* Expression in Differentiating C2C12 Cells

**Figure 3:**
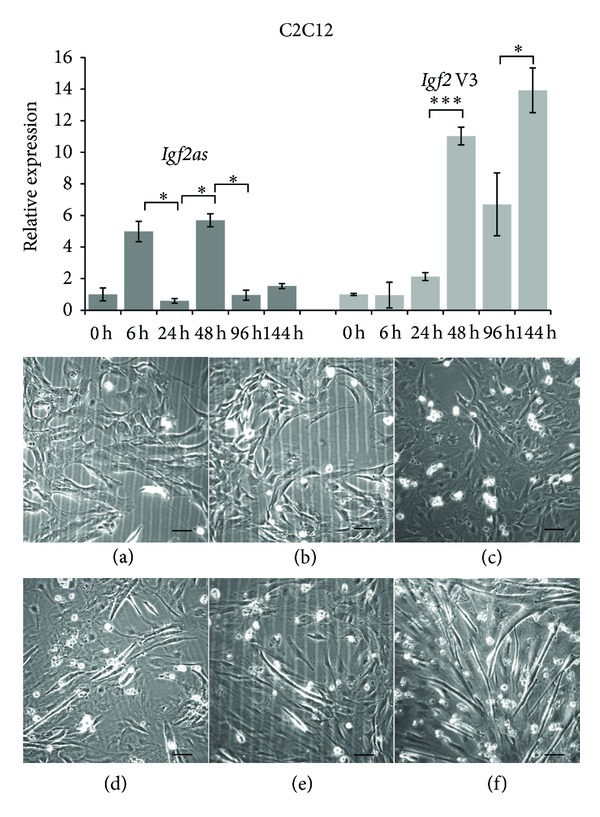
Quantification of *Igf2* and *Igf2as* expression during C2C12 myoblast differentiation. The C2C12 cells were maintained in differentiation media (DM) during a total time of 144 h. In the graph V3 and *Igf2as* correspond to *Igf2* variant 3 and *Igf2as *transcripts, respectively. The results are presented in relative expression. Each bar represents the mean of cells from three culture flasks each quantified in triplicate. The standard deviation for each time point is indicated. The images of C2C12 murine myoblast cells correspond to (a) C2C12 in growth media, (b) C2C12 after 6 h, (c) 24 h, (d) 48 h, (e) 96 h, and (f) 144 h in DM. The scale bar is indicated (30 *μ*m). *P < 0.05 and ***P < 0.001.

We used C2C12 myoblast cells differentiation to monitor the sense/antisense *Igf2/Igf2as* gene pair for potential interactions between the two partially complementary transcripts. Cells were differentiated by adding horse serum and the gene pair quantified at 0 h up to 144 h corresponding to 6 days (Figure 3). *Igf2* variant 3 expression levels augmented significantly during differentiation. *Igf2* variants 1 and 2 as well as Pm transcripts were expressed at very low levels (data not shown). However, at 144 h *Igf2* variant 1 peaked for unknown reason (data not shown). The low expression of these *Igf2* variants did not allow conclusive interpretation. Interestingly, *Igf2as* gene expression did not follow the increasing expressions trend of *Igf2* variant 3 during differentiation of C2C12 cells. *Igf2as *and* Igf2* expression increased significantly between 24 h and 48 h coinciding with the start of myotube formation.

### 3.3. *Igf2* and *Igf2as* Expressions in ΔDMR1-U2 and Wild-Type Mice

**Figure 4:**
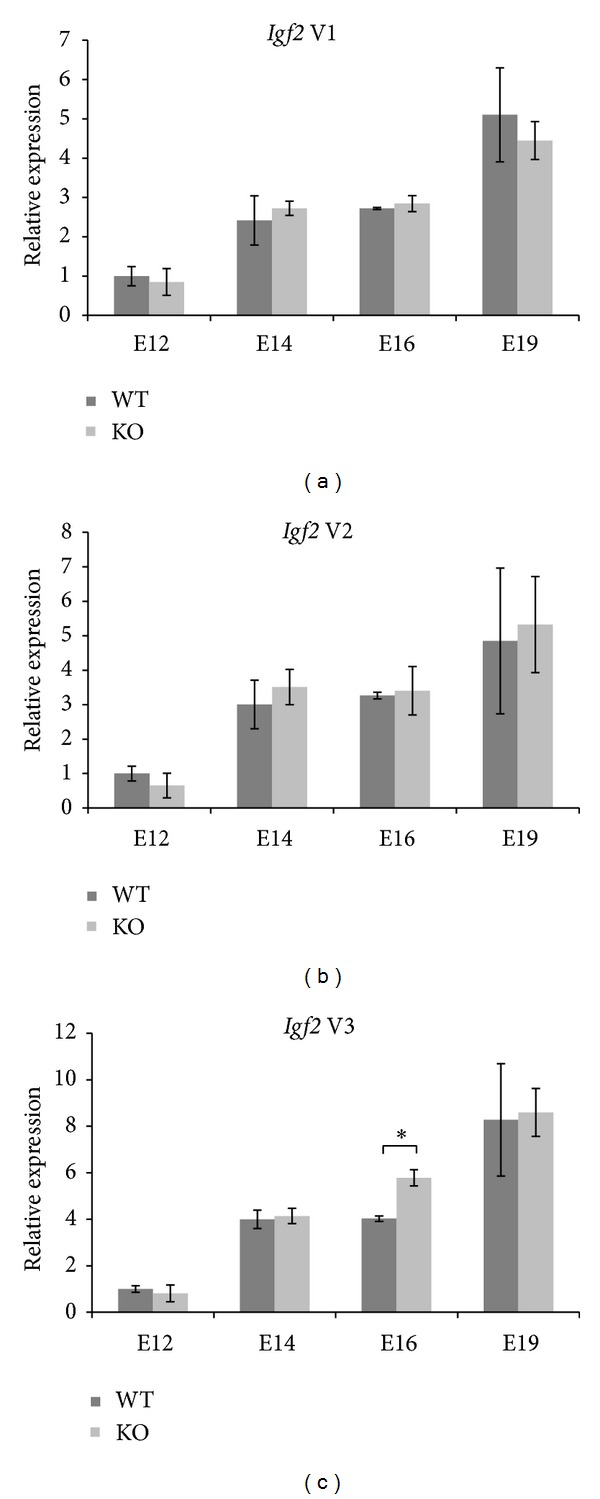
Quantification of *Igf2* variants expression levels in wild-type and ΔDMR1-U2 knockout placenta. Quantification of *Igf2* transcripts performed in wild-type (WT) and knockout (KO) placentas at different embryonic stages (E12, E14, E16, and E19). *Igf2* variants 1 (a), 2 (b), and 3 (c) were measured by qPCR. The results are expressed in relative expression. Each bar represents the mean of three samples each quantified in triplicate. The standard deviation is indicated by error bars. *P < 0.05.

**Figure 5:**
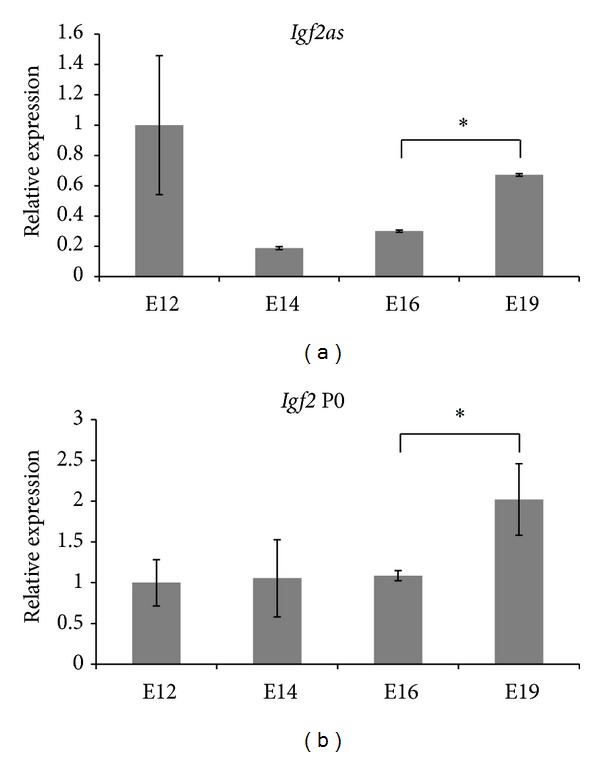
Quantification of *Igf2* P0 and *Igf2as *expression levels in wild-type placentas. Quantifications of *Igf2* P0 (P0) and *Igf2as* transcripts levels in wild-type placenta (WT) at different embryonic stages (E12, E14, E16, and E19). The results are expressed in relative expression. Each bar represents the mean of three samples analyzed in triplicate. The standard deviation for each bar is indicated by error bars. *P < 0.05.

We measured *Igf2* and *Igf2as* expressions in placentas of ΔDMR1-U2 mice and their wild-type litter mates (Figure 4). We found no differences in the expression of *Igf2* variants 1–3 between mutant and wild-type placentas except for *Igf2* variant 3 at E16, which was more highly expressed in the knockout placentas (Figure 4(c)). *Igf2as* expression levels in wild-type placenta significantly increased from E16 to E19 (Figure 5). The expression levels of *Igf2 *P0 in wild-type placenta were constant during E12 to E16 and augmented significantly from E16 to E19 (Figure 5). In ΔDMR1-U2 placentas no *Igf2as* and, as expected, no P0 transcripts were detected.

## 4. Discussion

The aim of this study was to further contribute to ascertaining the function of *Igf2as*. We studied *Igf2* and *Igf2as* gene expression in different tissues during development, in differentiating C2C12 cells, and in placenta of a ΔDMR1-U2 deletion mouse model. All *Igf2* variants and *Igf2as* followed a similar expression pattern in different tissues during development indicating a common regulation. In brain, we found a more continuous downregulation of *Igf2* and *Igf2as* transcription compared to distinct downregulation of these genes in adult liver and muscle. Most relevant is, however, that we did not find any consistent evidence for an interaction between *Igf2* and *Igf2as* transcripts although they are both present in the cytoplasm and contain complementary nucleotide sequences [5]. Additionally, we observed that the lack of *Igf2as *transcripts in placenta did not produce significant changes in *Igf2* variants expression levels compared to wild type with the exception for *Igf2* variant 3 at E16. *Igf2* variant 3 does not contain complementary sequences which could directly interact with *Igf2as* transcripts to form double-stranded RNA molecules. In addition variant 3 expression at E12, E14, and E19 was not different compared to wild-type mice and thus the interpretation of this event remains elusive. The comparison of *Igf2* and *Igf2as* expression between ΔDMR1-U2 and wild-type mice argues against the hypothesis that *Igf2as* transcripts have a role in *Igf2* gene regulation. The increase of *Igf2* variant 3 at E16 in mutant mice may have compensatory effect on *Igf2* protein content in labyrinthine trophoblast challenging the notion that the ΔDMR1-U2 phenotype is solely caused by the lack of P0 transcripts [7, 10]. During C2C12 differentiation, we observed different expression patterns for *Igf2 *variant 3 and *Igf2as* expressions arguing against an interaction between the two genes. Noteworthy is the increased expression of *Igf2 *variant 3 and *Igf2as* transcripts between 24 h and 48 h of differentiation when myotubes start to form.

The knockout mouse carrying the ΔDMR1-U2 deletion was extensively studied in regard to placental nutrient supply and fetal demand [6, 7, 9–11]. Here, we argue that the previously observed effects of the placenta specific *Igf2* P0 transcript may be confounded with the effects of the putative protein-coding *Igf2as* gene. Our alternative or additional explanation for the observed ΔDMR1-U2 deletion effects is therefore the lack of a functional *Igf2as* gene. The knockout of paternally expressed genes results in IUGR [13]. *Igf2as *is an imprinted and paternally expressed gene and therefore the IUGR phenotype described in ΔDMR1-U2 mice agrees with common functions of paternally expressed genes [13]. Assuming that the lack of a functional *Igf2as* gene contributes to the phenotype of IUGR, expressed as smaller placentas and lower birth weight, it might have similar functions as the *Igf2* gene. Interestingly, the recently described long-term behavioral effects found in ΔDMR1-U2 mice could also be, at least in part, due to the absence of *Igf2as* function [12]. This is suggested because we observed considerable *Igf2as* expression in brain during development. We think that the presented results indicate that previous studies have to be interpreted in the light of potential *Igf2as* gene effects. In summary, *Igf2as* is most expressed during prenatal development followed by a pronounced downregulation after birth in most tissues similar to the *Igf2* gene. In brain, we found also highest *Igf2as* expression in early development with a gradual downregulation from embryo brains to adult brains.

## Figures and Tables

**Table 1:** Primers pairs used for genotyping. Forward (F) and reverse (R) primers are indicated. WT refers to the RT-PCR product size in wild-type samples and KO refers to the product size in knockout ΔDMR1-U2 transgenic mouse.

Primer	Sequence (5′ > 3′)	Product
F_K0	AAGTTCCTCGGGTTGTAGGG	
R_K0	CTAGAGCAGTGTGGGGGTGT	582 bp (WT)
R_K0_control	TTGGCTAGAAGGCGAAAGAA	374 bp (KO)

**Table 2:** Primer pairs and probes used in TaqMan qPCR experiments. Forward (F) and reverse (R) primers are indicated.

Primer/probe	Sequence (5′ > 3′)	Product
F_Actb	GCTTCTTTGCAGCTCCTTCGT	
R_Actb	GCGCAGCGATATCGTCATC	
Probe_Actb	CCGGTCCACACCCGCCACC	71 bp
F_Gapdh	CGGCCGCATCTTCTTGTG	
R_Gapdh	TACGGCCAAATCCGTTCAC	
Probe_Gapdh	AGTGCCAGCCTCGTCCCGTAGACA	78 bp
F_Igf2 V1	CCGGCTTCCAGGTACCAAT	
R_Igf2 V1	GCAGCGATGCAGCACAAG	
Probe_Igf2 V1	ATGTTGGTGCTTCTCATCTCTTTGGCCTT	90 bp
F_Igf2 V2	GCCCTTCTCCTCCGATCCT	
R_Igf2 V2	ATGAGAAGCACCAACATCGACTT	
Probe_Igf2 V2	CGACCTTCGGCCTTGTGGTACCAA	98 bp
F_Igf2 V3	CCAGCCTTTTCCTGTCTTCATC	
R_Igf2 V3	CCATTGGTACCTGAAGTTGGGTAA	
Probe_Igf2 V3	TTCCAGCCCCAGCGGCCTC	68 bp
F_Igf2 P0	TGACTCCCCGGTCCTCTTT	
R_Igf2 P0	TGTTTTAATTACTCGTCCCCTGTACTC	
Probe_Igf2 P0	TCCACCGTCCGGGAACTTCAGG	84 bp
F_Igf2 Pm	TCGGAGAGAAGTAGGGTACCAATG	
R_Igf2 Pm	GGCGAAGGCCAAAGAGATG	
Probe_Igf2 Pm	AAGTCGATGTTGGTGCTT	77 bp
F_Igf2as MGB	TGCACCAAACTGATCAACACAA	
R_Igf2as MGB	AAAGGCAGGGTTCCAGATGA	
Probe_Igf2as MGB	CACACCAAAAATTCTTTC	62 bp
